# Food Safety and Invasive *Cronobacter* Infections during Early Infancy, 1961–2018

**DOI:** 10.3201/eid2605.190858

**Published:** 2020-05

**Authors:** Jonathan Strysko, Jennifer R. Cope, Haley Martin, Cheryl Tarr, Kelley Hise, Sarah Collier, Anna Bowen

**Affiliations:** Centers for Disease Control and Prevention, Atlanta, Georgia, USA

**Keywords:** Cronobacter, Cronobacter sakazakii, food safety, bacteria, meningitis/encephalitis, infants, powdered infant formula, neonatal sepsis

## Abstract

Invasive *Cronobacter* infections among infants are associated with severe neurologic disabilities and death. Early *Cronobacter* reports typically featured hospitalized and preterm infants and recognized contaminated powdered infant formula (PIF) as a transmission vehicle. To clarify recent epidemiology, we reviewed all cases of bloodstream infection or meningitis among infants that were reported to the Centers for Disease Control and Prevention and in the literature (1961–2018; n = 183). Most infants were neonates (100/150 [67%]); 38% (42/112) died, and 79% (81/102) had reported recent PIF consumption. In the final quarter of the study period (2004–2018), case counts were significantly higher (global average 8.7 cases/year); among US cases, significantly higher proportions occurred among full-term (56% [27/48]) and nonhospitalized (78% [42/54]) infants. PIF contamination, most commonly from opened containers, was identified in 30% (21/71) of investigations. Our findings reaffirm the need to promote safer alternatives for infant feeding, particularly among neonates.

## Abstract 2

Contaminated powdered infant formula from opened containers is the most commonly identified transmission vehicle.

*Cronobacter* species are gram-negative bacteria known to cause severe and often life-threatening infections in infants. Invasive *Cronobacter* infections in infants, including bloodstream infections and meningitis (most commonly caused by *C. sakazakii*), can result in neurologic disability, as well as death; reported case-fatality rates are as high as 40% (*1*). Beginning in 1961, reports of invasive *Cronobacter* infections historically described predominantly hospitalized and preterm infants (*2*,*3*). The identification of *Cronobacter* in association with several cases of necrotizing enterocolitis among infants reinforced its association with hospitalized infants born prematurely (*4*). However, previously healthy full-term infants are also known to become infected; infants born at more advanced gestational ages might be at greater risk than early preterm infants for having *Cronobacter* meningitis, as opposed to isolated bloodstream infection (*1*).

Because reporting is not mandatory in most countries (and in most of the United States), the true incidence of invasive infant *Cronobacter* infections is unknown. Estimates from laboratory-based surveillance in the United States suggest that ≈18 infant cases of invasive *Cronobacter* infection (0.49 cases/100,000 infants) occur annually (*5*). In 2008, the World Health Organization (WHO) reported the yearly incidence to be at least 0.14/100,000 infants in the Philippines and 1.76/100,000 infants in England and Wales, although these are thought to be underestimates (*6*).

*Cronobacter* spp. can withstand desiccation in dried foods like powdered infant formula (PIF) and are known to thrive in reconstituted formula (*7*). Linked to outbreaks among hospitalized neonates in the 1980s, contaminated PIF has been identified as the transmission vehicle in nearly all *Cronobacter* infections for which a source was found (*3*,*8*–*10*). The most recently identified US outbreak of *Cronobacter* infections linked to intrinsic contamination of a formula product (i.e., *Cronobacter* isolated from sealed formula containers) occurred in 2001 at a Tennessee hospital (*11*). That outbreak helped prompt the US Food and Drug Administration (FDA) in 2002 to discourage the use of PIF in neonatal intensive care settings unless there is no alternative available (*12*,*13*). For hospitalized neonates, FDA recommended ready-to-feed (RTF) liquid formula, which is considered sterile until opened. WHO issued broader recommendations aimed at preventing *Cronobacter* transmission through hygienic PIF reconstitution and storage practices, emphasizing the importance of hand hygiene and advising that caregivers reconstitute PIF with water heated to >70°C (*14*). In 2014, FDA issued quality control standards aimed at safer PIF production, including requiring manufacturers to routinely test for *Salmonella* and *Cronobacter* before distribution (*15*).

In 2016, the US Centers for Disease Control and Prevention (CDC) reported a case of invasive *Cronobacter* infection linked to contaminated expressed human breast milk (EBM) and the associated breast pump (*16*). After that case, 2 additional EBM-associated cases were reported in the literature, including 1 in a full-term neonate (*17*,*18*). To characterize recent epidemiology in light of regulatory actions, enhanced surveillance and preventive efforts, and newly described modes of transmission, we analyzed all cases of invasive *Cronobacter* infection among infants that were reported to the CDC and documented in the literature.

## Methods

We defined an invasive case as isolated bacteremia (*Cronobacter* isolated from blood) or meningitis (*Cronobacter* isolated from cerebrospinal or brain abscess fluid, with or without bacteremia) in an infant (<12 months of age). We excluded cases of isolated *Cronobacter* urinary tract infection and *Cronobacter-*associated necrotizing enterocolitis from this analysis. We searched for invasive cases reported since the first published reports (1961–2018) (*2*). The *Cronobacter* genus was formerly known as the single species *Enterobacter* *sakazakii*. Therefore, we used the subject heading terms “*Cronobacter*” or “*sakazakii*” in combination with “newborn,” “infant,” or “neonate” to conduct a literature search of the Medline, Embase, CINAHL, Scopus, and Cochrane Libraries and review associated bibliographies. In addition, we included previously unpublished cases from CDC case consultations, cases reported in the 2008 Food and Agriculture Organization of the United Nations (FAO) and WHO meeting notes summarizing the international call for data on *Cronobacter*, and the PubMLST database, an internet-based repository of bacterial isolate genetic sequences (*6*,*19*). PubMLST submission profiles occasionally report clinical and demographic information, which led to the identification of additional cases. We de-duplicated redundant cases reported in multiple data sources using information provided on patient age, location, and illness onset dates.

We used “community-onset cases” and “cases among non-hospitalized patients” interchangeably to signify that symptom onset occurred outside the hospital. We defined preterm birth as <37 weeks (early preterm, <32 weeks; late preterm, >32 to <37 weeks) and full-term birth as >37 weeks estimated gestational age (EGA). We defined a neonate as an infant <28 days of age. For PIF, we used any commercially manufactured PIF product, including powdered human milk fortifier. (In the United States, powdered formulas, including follow-on formulas, are classified as PIF if they are intended for use among infants).

In addition to reviewing clinical information and feeding histories for each case, we reviewed results of available case investigations. Although there was no standard definition for food consumption in relation to disease onset across data sources, CDC’s reporting convention included any consumption within 7 days before symptom onset. *Cronobacter* infections are not nationally notifiable in the United States, but state and local health departments encountering new *Cronobacter* cases may contact CDC to request clinical consultation and submit clinical isolates, food, and environmental samples for laboratory testing. When possible, FDA tests PIF or other products from sealed containers of the same lots fed to the infant to assess whether contamination occurred during production. CDC used pulsed-field gel electrophoresis (PFGE) to assess similarity between clinical, food, and environmental isolates using 2 restriction enzymes, *Xba*1 and *Spe*1. However, PFGE might have limited capacity to differentiate between genetically unrelated strains within the same clonal complex. In other investigations, whole-genome sequencing and multilocus sequencing typing has offered more precise determinations of genetic similarity.

We considered cases to be outbreak-associated if the clinical isolate’s PFGE pattern was indistinguishable from that of another case (invasive, noninvasive, or colonized). Alternatively, cases could be designated as outbreak-associated if they were detected in proximity with other cases both temporally (within 6 months) and spatially (in the same home or hospital). Defining the reporting period as 1961–2018, we compared reporting rates during the final quarter (2004–2018) with those during the preceding 3 quarters. We performed descriptive analysis using SAS software version 9.4 (https://www.sas.com), characterizing trends in annual reporting with negative-binomial regression, and comparing groups using χ^2^, *t*-tests, and Wilcoxon signed-rank tests.

## Results

We identified 183 unique infants who met the case definition: 66 described in the literature, 61 from CDC case consultations, 53 from the WHO/FAO report, and 3 from PubMLST. Cases were reported from 24 countries across 6 continents (Table 1; Appendix) Global annual reporting was significantly higher during the final quarter of the study period, increasing from a mean of 1.2 cases/year before 2004 to 8.7 cases/year from 2004 on (p<0.01). More than two thirds (130/183) of the cases were reported in the final quarter, when the proportion of outbreak-associated cases was significantly lower both in the United States and internationally (Table 2).

**Table 1:** Numbers of reported invasive *Cronobacter* infections among infants, by country, 1961–2018

Country	No. cases reported, n = 183	Appendix reference*
Argentina	3	(*16*)
Australia	1	(*10*)
Belgium	2	(*3**,**14*)
Brazil	8	(*11**,**17**–**19*)
Canada	3	(*20**–**22*)
China	3	(*23**,**24*)
Denmark	1	(*25*)
France	5	(*4**,**15**,**26*)
Germany	1	(*27*)
Greece	1	(*28*)
Iceland	3	(*6*)
India	2	(*29*)
Israel	4	(*12*)
Japan	2	(*4**,**13*)
Kenya	1	(*30*)
Netherlands	8	(*2*)
New Zealand	1	(*31*)
Philippines	16	(*4*)
Portugal	1	(*32*)
Romania	1	(*33*)
Spain	1	(*34*)
Switzerland	1	(*35*)
United Kingdom	35	(*1**,**4*)
United States	79	(*5**,**7**,**8**,**9**,36–48*)

*A full list of reviewed manuscripts is provided in the Appendix.

**Table 2:** Characteristics of 183 infants with invasive *Cronobacter* infection, overall and by clinical syndrome type, geographic location, and reporting period, 1961–2018*

Characteristic*	Overall	Syndrome type			United States			Outside United States	
		Bacteremia	Meningitis		1st–3rd quarters, 1961–2003	4th quarter, 2004–2018		1st–3rd quarters, 1961–2003	4th quarter, 2004–2018
Sex									
F	50/103 (49)	20/35 (57)	30/68 (44)		7/17 (44)	24/49 (49)		9/19 (53)	9/18 (50)
M	53/103 (51)	15/35 (43)	38/68 (56)		10/17 (56)	25/49 (51)		10/19 (47)	9/18 (50)
Cesarean delivery	33/58 (57)	13/19 (68)	20/39 (51)		2/5 (40)	20/24 (48)		7/10 (70)	0/1 (100)
Died	42/112 (38)	13/46 (28)	29/66 (44)		4/21 (19)	9/42 (21)		14/27 (52)	14/21 (67)
Neonatal onset	100/150 (67)	36/55 (65)	64/95 (67)		16/24 (67)	40/55 (73)		19/20 (95)	16/20 (80)
Median onset age, d	13 (7–28)	17 (9–33)	11 (6–22)		20 (10–35)	16.5 (11–30)		5 (4–14)	**11.5 (5–60)**
Median EGA, wks	36 (31–40)	31 (27–39)	**36 (32.2–39)**		32 (30–36)	**37 (32.2–39)**		37 (32–40)	**31 (27.7–40)**
Median birthweight, g	2,155 (1,400–2,948)	1,626 (1,044–2,845)	**2,514 (1,843–2,989)**		1,475 (900–2,477)	**2,594 (1,815–3,118)**		2,277 (1,545–2,775)	**1,570 (1,269–1,980)**
Community onset	57/119 (48)	32/48 (67)	**41/71 (58)**		8/18 (44)	**42/54 (77)**		2/25 (8)	5/22 (23)
Full-term birth	41/100 (41)	11/33 (33)	**30/67 (45)**		4/18(22)	**27/48 (57)**		10/20 (50)	**1/15 (7)**
Late preterm birth	29/100 (29)	4/33 (12)	**25/67 (37)**		5/18 (27)	13/48 (27)		6/20 (30)	6/15 (40)
Early preterm birth	30/100 (30)	18/33 (55)	**12/66 (18)**		9/18 (50)	**9/48 (19)**		4/20 (20)	**8/15 (53)**
Outbreak-associated	33/125 (26)	11/47(23)	22/78(28)		4/21 (19)	**2/53 (4)**		20/29 (69)	**7/22 (32)**

*Values are no. (%) or median (interquartile range). Neonatal onset defined as <28 d after birth; full-term birth was ≥37 weeks EGA; late preterm birth, 32–37 weeks EGA; early preterm birth, <32 weeks EGA. Outbreak-associated was defined as a clinical isolate’s pulsed-field gel electrophoresis pattern indistinguishable from that of another case (invasive, noninvasive, or colonized) or if detected in proximity with other cases both temporally (within 6 mo) and spatially (in the same home or hospital). Bold type indicates statistical significance (p<0.05) between reporting periods or syndrome types. EGA, estimated gestational age.

Among 79 US cases, most (61 [77%]) were reported to CDC; 15 cases were described in the literature only, most (13 [87%]) published before 2004. The proportion of cases among nonhospitalized US infants increased significantly, from 44% (8/18) before 2004 to 78% (42/54) of cases for 2004–2018 (p*<*0.01 by χ^2^ test) (Figure). The proportion of cases reported among full-term US infants was also higher during this period; 22% (4/18) before 2004, compared with 56% (27/48) for 2004–2018 (p *=* 0.01 by χ^2^ test) (Table 2). In contrast, outside the United States, a minority of cases occurred among nonhospitalized [15% (7/47)] or full-term [31% (11/47)] infants.

**Figure:**
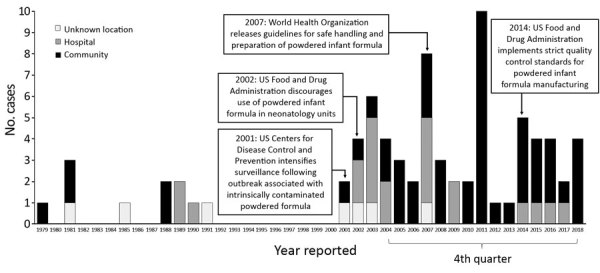
Reported invasive *Cronobacter* infections among infants, United States, 1979–2018, by location of patient at the time of symptom onset (n = 79). (The first case of invasive infant *Cronobacter* infection in the United States was reported in 1979 [*13–15*].)

### Clinical Characteristics and Outcomes

Overall, 116 (63%) infants had meningitis and 67 (37%) had isolated bacteremia. Compared with patients with isolated *Cronobacter* bacteremia, patients with *Cronobacter* meningitis were significantly more likely to have experienced onset outside the hospital (58% [41/71] vs. 33% [16/48]; p<0.01) and were typically born closer to term (median 36 weeks EGA vs. 31 weeks EGA; p = 0.01) (Table 2). The median EGA of patients overall was 36 (IQR 31–40) weeks. Nearly all infants (95% [140/148]) became ill within the first 2 months after birth, 67% (100/150) in the neonatal period; median age at symptom onset was 13 (IQR 7–28) days.

Immunocompromising conditions other than prematurity were reported in only 1 patient, a 10-month-old infant with severe combined immunodeficiency. Concurrent conditions (other than common prematurity-related complications) were also infrequently reported but included gastrointestinal anomalies (n = 4 [2%]), congenital heart disease (n = 3 [2%]), and a neural tube defect (n = 1). Medical interventions before illness onset included enteral tube feeding (n = 16 [9%]), mechanical ventilation (n = 10 [5%]), and recent antimicrobial drug therapy (n = 9 [5%]).

Case-fatality overall was 38% (42/112) and did not change significantly over time (Table 2). Among survivors, complications included cerebral abscess or infarction (27% [18/67]) and hydrocephalus (16% [11/67]).

### Feeding Practices and Case Investigations

Feeding histories were described for 102 infants. Eighty-one (79%) reported recent consumption of PIF, with or without other supplemental feeding types, and 48 (47%) consumed PIF exclusively. Exclusive consumption of liquid infant formula was reported in 4 cases. Among 10 infants reported to consume only breastmilk in the 7 days before symptom onset, 5 received expressed breastmilk; reports did not clarify whether the breastmilk was expressed in the remaining 5 cases.

Source investigations were conducted for 71 (39%) cases. At least one contaminated environmental or food isolate was identified for 31 (44%) of these cases, and PIF contamination was identified in 30% (21/71) of investigations. Among 17 cases outside of the United States that had source investigations conducted, 14 (82%) had a transmission vehicle identified; 11 (79%) cases were linked to contaminated PIF (6 from sealed containers, 5 from opened containers), 2 (14%) with blenders used to mix PIF, and 1 (7%) with contaminated EBM/breast pump (*4*,*9*,*17*,*20*–*25*).

Among 54 CDC-supported source investigations, >1 contaminated environmental or food isolate was identified for 17 (32%) investigations, each linked to a single case (Table 3). *Cronobacter* was isolated from 10 (22%) of the 46 opened PIF containers tested; 5 (50%) of these isolates were obtained during 2004–2018. CDC identified *Cronobacter* from opened PIF containers in 4 additional instances during 2004–2018; of these, 3 were associated with noninvasive cases and 1 was associated with a noninfant. All 4 PFGE patterns were indistinguishable from corresponding clinical isolates. FDA did not identify contaminated PIF from lot-matched sealed containers associated with clinical cases during 2001–2018; however, testing was not performed for all cases.

**Table 3:** Results of investigations supported by the Centers for Disease Control and Prevention for 54 cases of invasive *Cronobacter* infections among infants, United States, 1989–2018*

Sample type	Sample type tested	Sample yielded *Cronobacter*	Sample isolate indistinguishable from clinical isolate†
PIF from opened containers	46/52 (87)	10/46 (22)‡	6/7 (86)
Breast pump collection kit/pump-expressed breastmilk	5/52 (9)	2/5 (40)	1/2(50)
Water from opened bottle	16/52 (27)	2/16 (13)	1/1 (100)
Environmental surfaces	17/52 (31)	6/17 (35)§	4/6 (67)

*Values are no. positive/no. tested (%). Results from first source investigation in the United States was reported in 1989 (*8*). †Comparison made using pulsed-field gel electrophoresis. ‡*Cronobacter* contamination was identified in powdered infant formula during investigations conducted during 1989–2017. §Of 6 cases that yielded an isolate from an environmental surface, 4 were from kitchen sink surfaces, 1 from a pacifier, and 1 from a bottle nipple.

PIF preparation practices were recorded for only 4 cases in which *Cronobacter* was isolated from an opened PIF container. All caregivers reported proper hand hygiene; none reported reconstituting PIF using the WHO-recommended method of heating water to >70°C. In addition, evidence of environmental contamination was found during investigations of 6 cases: sink surfaces (n = 4), a bottle nipple (n = 1), and a pacifier (n = 1). Two cases involved contaminated EBM/breast pumps, and 2 cases involved contaminated bottled water used to reconstitute PIF. Among *Cronobacter* isolates identified from contaminated food or environmental samples, PFGE patterns were indistinguishable from corresponding clinical isolates 81% (13/16) of the time.

## Discussion

Our findings provide continued evidence that invasive *Cronobacter* infections disproportionately affect infants in the neonatal period and are associated with high mortality. Early *Cronobacter* reports often featured hospitalized and preterm infants, but our findings suggest a rising majority of cases occurring among nonhospitalized and full-term infants in the United States. Contaminated PIF from opened containers is the most commonly identified transmission vehicle.

Reports of invasive infant *Cronobacter* infections appear to have increased globally, despite the lack of mandatory reporting. It is unclear whether the true incidence increased; this reporting increase might be because of more precise microbiologic identification, increased interest following publicized outbreaks and the WHO call for cases, and greater awareness about the larger public health implications (*6*,*26*). In addition, there was no name for *Cronobacter* spp. during the first quarter of reporting; early isolates were identified as *Cronobacter* spp. retrospectively. *Enterobacter sakazakii* was named as a species in 1980, and *Cronobacter* was proposed as a genus in 2007 (*27*). It is possible that the evolving nomenclature and identification methods may have resulted in both missed cases and inclusion of infections misclassified as *Cronobacter.*

*Cronobacter* reports outside the United States still predominantly feature hospitalized and preterm infants, whereas recent cases reported within the United States reflect higher proportions of cases among full-term, nonhospitalized infants. The reasons for these differences are likely multifactorial. Because we relied on published reports for cases among infants outside the United States, the characteristics of this group could have been influenced by a publishing bias; patients cared for in academic settings with advanced care capability might be the most likely to be detected and reported in the literature. In addition, outbreaks are more likely to be detected when cases occur in proximity, such as in neonatal intensive care units where most infants are preterm; although proportions of outbreak-associated cases declined globally in the final quarter, nearly one third of cases outside the United States were known to be associated with an outbreak (as opposed to 4% of US cases during that period). Likewise, the significant rise in cases among US full-term and nonhospitalized infants might be an artifact of surveillance changes during the period we examined; starting in 2001, CDC called for cases to be voluntarily reported in the United States, which likely contributed to higher proportions of sporadic cases being reported. Differential changes in risk exposure might also contribute to higher proportions of cases among full-term infants. In the United States, the 2002 FDA recommendation discouraging the use of PIF among hospitalized infants has likely helped prevent some cases among hospitalized and preterm infants; however, PIF continues to be used in neonatology units throughout much of the world (*20*).

High-risk groups for invasive *Cronobacter* infection include infants <2 months of age and infants born prematurely (even as reports of cases among full-term infants in the United States are becoming more common). Other clinical risk factors, however, are not straightforward. Immunocompromising and other concurrent conditions other than prematurity were not frequently reported and are not thought to be major drivers of disease acquisition in infants; reports of medical devices/interventions were also not common. Like previous reports, this analysis found *Cronobacter* meningitis was more common in full-term and late preterm infants, whereas *Cronobacter* bacteremia was more common in early preterm infants (*1*). The reason for this difference is unclear; however, timely initiation of empiric antimicrobial drug treatment among inpatients undergoing clinical monitoring could prevent invasion of bacteria beyond the blood–brain barrier (*7*).

PIF consumption was reported in most cases, and PIF from opened containers is the most commonly identified transmission vehicle. Even with small amounts of the remaining product available for testing, 10 (22%) PIF containers yielded *Cronobacter* during US investigations. More than half of these *Cronobacter*-contaminated containers were identified during the final quarter of the analytic period, as recently as 2017. CDC also identified *Cronobacter*-contaminated PIF from opened containers being used to feed 4 additional noninfant children or infants with noninvasive infections during 2004–2018, which were not included in this analysis (CDC, unpub. data). Finding *Cronobacter* in opened PIF containers does not prove that the PIF was intrinsically contaminated, as opposed to being contaminated after the container was opened; aside from the 2001 outbreak in a Tennessee hospital, FDA did not identify contaminated PIF from lot-matched sealed containers during this time period. Still, *Cronobacter* contamination during manufacturing remains a possibility; heterogeneous contamination might not be detected through even the most sensitive sampling schemes conducted by FDA and manufacturers (*27*).

Because PIF remains the most commonly identified transmission vehicle, prevention efforts should focus on minimizing the risk of PIF contamination through regulatory, engineering, and behavioral efforts, and promoting safer alternatives to PIF, particularly among infants 0–2 months of age. First, sustained focus is needed on identifying and maintaining effective quality control measures during PIF manufacturing. In keeping with regulatory standards, heat-labile nutrients are added to PIF after pasteurization, making it vulnerable to contamination during production, particularly with organisms like *Cronobacter*, which can persist for long periods in dry environments (*28*). The implementation of FDA’s 2014 quality control standards for safer PIF production was a crucial step toward preventing the distribution of intrinsically contaminated PIF. However, recent outbreaks of infant salmonellosis linked to contaminated PIF produced in Spain in 2010 and France in 2017 highlight the ongoing risk for PIF contamination occurring during production and the value of environmental monitoring for potential contamination (*29*,*30*).

Even if contamination does not occur during production, PIF can easily become contaminated once containers are opened and exposed to the environment. Although vigilant adherence to best hand-hygiene practices is necessary when preparing, handling, and storing PIF, engineering solutions, which remain a cornerstone of public health preventive efforts, are also needed. Redesigning PIF packaging with the aim of minimizing environmental exposure and contact with contaminated surfaces could help prevent transmission of *Cronobacter* and other pathogens (*31*).

Healthcare providers, lactation consultants, and nutritionists play vital roles in supporting and educating caregivers about the risks of PIF contamination (*32*). They can help communicate that PIF is not a sterile product, while providing education and support for caregivers who choose to use PIF. The 2007 WHO recommendations to reconstitute PIF with water heated to >70°C should reduce pathogen load in reconstituted PIF, but these recommendations have not been universally adopted because of concerns about the potential effect on heat-sensitive nutritional components (including probiotics), impracticality, and burn risks (*12*). One report also suggested that the WHO-recommended method might not effectively kill all strains of *Cronobacter* (*33*). With these concerns in mind, promotion of safer alternatives to PIF, particularly for infants in the neonatal period, is needed.

Safer alternatives to PIF include direct breastfeeding, feeding with breastmilk that has been expressed safely, and feeding with liquid formula that has been safely handled and stored. Although the number of *Cronobacter* cases among infants who consumed PIF far outnumbered those among infants who exclusively received breastmilk, emerging reports of cases linked to contaminated EBM raise concern about hygiene practices during expression and handling of breastmilk. Comprehensive support can help caregivers to adhere to best practices. CDC offers guidance for proper breast pump hygiene, advising caregivers to wash hands thoroughly before handling pump equipment and to take apart and clean breast pump kits in the dishwasher or by hand with soap and water with a dedicated basin and brush after every use; air dry them on a clean surface in a protected area; and sanitize them at least daily by boiling, steaming, or using a dishwasher’s sanitize cycle.

The associated costs and required system supports should be taken into account when weighing the costs and benefits of PIF and PIF alternatives. RTF formula is more expensive than PIF; 2012 comparisons estimated that milk-based RTF formula cost US $0.84 (29%) more per day than milk-based PIF (*12*). Increased support for parental leave and onsite lactation facilities at places of employment will also make direct breastfeeding and safe breastmilk expression easier during critical exposure periods for *Cronobacter* and other infant illnesses.

The cases in which no source/vehicle was identified, as well as the cases occurring among infants who did not consume PIF or EBM, suggest that alternate modes of transmission are possible. Vertical transmission, although theoretically possible, is probably rare; only 4 patients experienced symptom onset in the first 48 hours after birth. *C. malonaticus* has been isolated from a breast abscess, but transmission during direct breastfeeding has never been reported (*34*). Contaminated environmental specimens suggest that fomite transmission could also occur, although the source of the contamination is unclear. Two cases involving *Cronobacter*-contaminated water (Table 3) highlight that even bottled and previously boiled water used to reconstitute PIF could become contaminated once opened and exposed to the environment, particularly if it does not contain residual chlorine.

This study has limitations. The changes in surveillance we describe might have affected the trend analyses, and because reporting *Cronobacter* cases is largely not mandatory, the analysis might have been influenced by a severity bias. Additional limitations include incomplete reporting of clinical characteristics, laboratory confirmation, feeding histories, and long-term outcomes. In some cases, clinical isolates were discarded before a public health laboratory could confirm the diagnosis. Other cases might have been excluded or the clinical syndrome misclassified if cultures were taken after initiation of antimicrobial drug therapy. Limitations of case investigations include that many food and environmental specimens were unavailable at the time of the investigation. Finally, the *Cronobacter* isolates included in this analysis were compared using PFGE, which might have limited capacity to differentiate between genetically unrelated strains within the same clonal complex. In other investigations, whole-genome sequencing and multilocus sequencing typing have offered more precise determinations of genetic similarity (*35*).

Considering the potential for severe outcomes and far-reaching policy implications, jurisdictions may consider making invasive *Cronobacter* infections among infants a reportable condition. Mandatory reporting with standardized reporting procedures would help better characterize incidence, elucidate risk factors, promptly detect outbreaks, and inform prevention measures. We encourage public health officials to contact CDC when investigating invasive *Cronobacter* cases in infants.

Appendix Additional references regarding food safety and invasive *Cronobacter* infections in early infancy.
