# Supplementary material for: Food Safety and Invasive Cronobacter Infections during Early Infancy, 1961–2018
Source: Emerg Infect Dis. 2020 May;26(5):857–65. doi: 10.3201/eid2605.190858 (PMC7181934; doi:10.3201/eid2605.190858)
Supplement: Appendix — Additional references regarding food safety and invasive Cronobacter infections in early infancy. [file 19-0858-Techapp-s1.pdf]

Article DOI: https://doi.org/10.3201/eid2605.190858

# Food Safety and Invasive *Cronobacter* Infections during Early Infancy, 1961–2018

## Appendix

### References

1. Urmenyi AM, Franklin AW. Neonatal death from pigmented coliform infection. Lancet. 1961;1:313–5. https://doi.org/10.1016/S0140-6736(61)91481-7

2. Muytjens HL, Zanen HC, Sonderkamp HJ, Kollée LA, Wachsmuth IK, Farmer JJ III. Analysis of eight cases of neonatal meningitis and sepsis due to *Enterobacter sakazakii*. J Clin Microbiol. 1983;18:115–20. https://doi.org/10.1128/JCM.18.1.115-120.1983

3. van Acker J, de Smet F, Muyldermans G, Bougatef A, Naessens A, Lauwers S. Outbreak of necrotizing enterocolitis associated with *Enterobacter sakazakii* in powdered milk formula. J Clin Microbiol. 2001;39:293–7. https://doi.org/10.1128/JCM.39.1.293-297.2001

4. Food and Agriculture Organization of the United Nations / World Health Organization. *Enterobacter sakazakii* (*Cronobacter* spp.) in powdered follow-up formulae: meeting report. 2008 [cited 2019 Feb 14]. https://www.who.int/foodsafety/publications/micro/MRA_followup.pdf

5. Simmons BP, Gelfand MS, Haas M, Metts L, Ferguson J. *Enterobacter sakazakii* infections in neonates associated with intrinsic contamination of a powdered infant formula. Infect Control Hosp Epidemiol. 1989;10:398–401. https://doi.org/10.2307/30144207

6. Biering G, Karlsson S, Clark NC, Jónsdóttir KE, Lúdvígsson P, Steingrímsson O. Three cases of neonatal meningitis caused by *Enterobacter sakazakii* in powdered milk. J Clin Microbiol. 1989;27:2054–6. https://doi.org/10.1128/JCM.27.9.2054-2056.1989

7. Noriega FR, Kotloff KL, Martin MA, Schwalbe RS. Nosocomial bacteremia caused by *Enterobacter sakazakiki* and *Leuconostoc mesenteroides* resulting from extrinsic contamination of infant formula. Pediatr Infect Dis J. 1990;9:447–9. https://doi.org/10.1097/00006454-199006000-00018

8. Centers for Disease Control and Prevention. *Enterobacter sakazakii* infections associated with the use of powdered infant formula—Tennessee, 2001. JAMA. 2002;287:2204–5.

9. Bowen A, Wiesenfeld HC, Kloesz JL, Pasculle AW, Nowalk AJ, Brink L, et al. Notes from the field: *Cronobacter sakazakii* infection associated with feeding extrinsically contaminated expressed human milk to a premature infant—Pennsylvania, 2016. MMWR Morb Mortal Wkly Rep. 2017;66:761–2. https://doi.org/10.15585/mmwr.mm6628a5

10. McMullan R, Menon V, Beukers AG, Jensen SO, van Hal SJ, Davis R. *Cronobacter sakazakii* infection from expressed breast milk, Australia. Emerg Infect Dis. 2018;24:393–4. https://doi.org/10.3201/eid2402.171411

11. Chaves CEV, Brandão MLL, Lacerda MLGG, Rocha CABC, Leone de Oliveira SMDV, Parpinelli TC, et al. Fatal *Cronobacter sakazakii* sequence type 494 meningitis in a newborn, Brazil. Emerg Infect Dis. 2018;24:1948–50. https://doi.org/10.3201/eid2410.180373

12. Block C, Peleg O, Minster N, Bar-Oz B, Simhon A, Arad I, et al. Cluster of neonatal infections in Jerusalem due to unusual biochemical variant of *Enterobacter sakazakii*. Eur J Clin Microbiol Infect Dis. 2002;21:613–6. https://doi.org/10.1007/s10096-002-0774-5

13. Teramoto S, Tanabe Y, Okano E, Nagashima T, Kobayashi M, Etoh Y. A first fatal neonatal case of *Enterobacter sakazakii* infection in Japan. Pediatr Int. 2010;52:312–3. https://doi.org/10.1111/j.1442-200X.2010.03030.x

14. Drudy D, Mullane NR, Quinn T, Wall PG, Fanning S. *Enterobacter sakazakii*: an emerging pathogen in powdered infant formula. Clin Infect Dis. 2006;42:996–1002. https://doi.org/10.1086/501019

15. Coignard B, Vaillant V, Vincent JP, Lefleche A, Mariani-Kurkdjian P, Bernet C, et al.. Severe infections of *Enterobacter sakazakii* in neonates who consumed powdered infant formula, France, October–December 2004 [in French]. Bulletin Epidemiologique Hebdomadaire. 2006;2–3:10–3.

16. Asato VC, Vilches VE, Pineda MG, Casanueva E, Cane A, Moroni MP, et al. First clinical isolates of *Cronobacter* spp. (*Enterobacter sakazakii*) in Argentina: characterization and subtyping by pulsed-field gel electrophoresis. Rev Argent Microbiol. 2013;45:160–4. https://doi.org/10.1016/S0325-7541(13)70018-X

17. Santos M, Pessoa da Silva CL, Sampaio J, Marangoni DV, Pinto M, Moreira BM. Infection and control of *Enterobacter sakazakii* sepsis outbreak in four hospitals in Rio di Janiero, Brazil. Infect Control Hosp Epidemiol. 2000;21:140.

18. Barriera ER, Costa de Souza D, de Freitas Gois P, Fernandes JC. *Enterobacter sakazakii* in a newborn: case report. [in Portuguese]. Pediatria. 2003;25:65–70.

19. Estrela GT, Silveira TH, De Campos FR, Pedigone MAMC, Brunherotti MAA, Pires RH. Microorganisms isolated in newborns in the specialized hospital assistance. Pediatr Pulmonol. 2016;51(Supplement 42):S53.

20. Pagotto FJ, Farber JM. *Cronobacter* spp. (*Enterobacter sakazakii*): advice, policy and research in Canada. Int J Food Microbiol. 2009;136:238–45. https://doi.org/10.1016/j.ijfoodmicro.2009.05.010

21. Ouchenir L, Renaud C, Khan S, Bitnun A, Boisvert AA, McDonald J, et al. The epidemiology, management, and outcomes of bacterial meningitis in infants. Pediatrics. 2017;140:e20170476. https://doi.org/10.1542/peds.2017-0476

22. Peebles ER, VanHooren TA, Gunz AC, Salvadori MI. Case 4: poor feeding and lethargy in a 32-day-old infant. Pediatr Rev. 2018;39:95. https://doi.org/10.1542/pir.2016-0159

23. Cui JH, Yu B, Xiang Y, Zhang Z, Zhang T, Zeng YC, et al. Two cases of multi-antibiotic resistant *Cronobacter* spp. infections of infants in china. Biomed Environ Sci. 2017;30:601–5.

24. Zeng H, Lei T, He W, Zhang J, Liang B, Li C, et al. Novel multidrug-resistant *Cronobacter sakazakii* causing meningitis in neonate, China, 2015. Emerg Infect Dis. 2018;24:2121–4. https://doi.org/10.3201/eid2411.180718

25. Joker RN, Norholm T, Siboni KE. A case of neonatal meningitis caused by a yellow enterobacter. Dan Med Bull. 1965;12:128–30.

26. Caubilla-Barron J, Hurrell E, Townsend S, Cheetham P, Loc-Carrillo C, Fayet O, et al. Genotypic and phenotypic analysis of *Enterobacter sakazakii* strains from an outbreak resulting in fatalities in a neonatal intensive care unit in France. J Clin Microbiol. 2007;45:3979–85. https://doi.org/10.1128/JCM.01075-07

27. Ries M, Harms D, Scharf J. Multiple cerebral infarctions in a premature baby with meningitis due to *Enterobacter sakazakii* leading to multicystic encephalomalacia [in German]. Klin Padiatr. 1994;206:184–6. https://doi.org/10.1055/s-2008-1046601

28. Arseni A, Malamou-Ladas E, Koutsia C, Xanthou M, Trikka E. Outbreak of colonization of neonates with *Enterobacter sakazakii*. J Hosp Infect. 1987;9:143–50. https://doi.org/10.1016/0195-6701(87)90052-1

29. Ray P, Das A, Gautam V, Jain N, Narang A, Sharma M. *Enterobacter sakazakii* in infants: novel phenomenon in India. Indian J Med Microbiol. 2007;25:408–10. https://doi.org/10.4103/0255-0857.37351

30. Piper JD, Mwarumba S, Ngari M, Mvera B, Morpeth S, Berkley JA. Invasive *Cronobacter* species infection in infants and children admitted to a rural Kenyan hospital with a high prevalence of malnutrition. Paediatr Int Child Health. 2018;38:198–202. https://doi.org/10.1080/20469047.2018.1446485

31. New Zealand Ministry of Health. Inquiry into actions of sector agencies in relation to contamination of infant formula with *Enterobacter sakazakii*. 2005 March [cited 2019 Feb 14]. http://www.moh.govt.nz/notebook/nbbooks.nsf/0/0a9db19cd852af71cc256fdb000e3d61/$FILE/enterobactersakazakii-report.pdf.

32. Lecour H, Seara A, Cordeiro J, Miranda M. Treatment of childhood bacterial meningitis. Infection. 1989;17:343–6. https://doi.org/10.1007/BF01650726

33. Marinescu AR, Crisan A, Lăzureanu V, Musta V, Nicolescu N, Laza R. *Cronobacter sakazakii* sepsis in varicella patient. Presented at: BMC Infectious Diseases Conference: 12th Scientific Days of the National Institute for Infectious Diseases "Prof Dr Matei Bals" and the 12th National Infectious Diseases Conference, Bucharest, Romania; November 23–25, 2016.

34. Aguirre Conde A, Pérez Legorburu A, Echániz Urcelay I, Hernando Zárate Z, Arrate Zugazabeitia JK. Neonatal sepsis due to *Enterobacter sakazakii* [in Spanish]. An Pediatr (Barc). 2007;66:196–7. https://doi.org/10.1016/S1695-4033(07)70336-3

35. PubMLST. Isolate z3032 (id:77) 2010 Jan 13 [cited 2019 Feb 14] https://pubmlst.org/bigsdb?page=info&db=pubmlst_cronobacter_isolates&id=77

36. Monroe PW, Tift WL. Bacteremia associated with *Enterobacter sakazakii* (yellow, pigmented *Enterobacter* cloacae). J Clin Microbiol. 1979;10:850–1. https://doi.org/10.1128/JCM.10.6.850-851.1979

37. PubMLST. Isolate FSL F6–049 (id:380). 2012 Jul 4 [cited 2019 Feb 19]. https://pubmlst.org/bigsdb?page=info&db=pubmlst_cronobacter_isolates&id=380

38. Adamson DH, Rogers JR. *Enterobacter sakazakii* meningitis with sepsis. Clin Microbiol Newsl. 1981;3:19–20. https://doi.org/10.1016/S0196-4399(81)80039-6

39. PubMLST. Isolate 1218 (id:1115). 2015 Feb 20 [cited 2019 Feb 14]. https://pubmlst.org/bigsdb?page=info&db=pubmlst_cronobacter_isolates&id=1115

40. Gallagher PG, Ball WS. Cerebral infarctions due to CNS infection with *Enterobacter sakazakii*. Pediatr Radiol. 1991;21:135–6. https://doi.org/10.1007/BF02015629

41. Willis J, Robinson JE. *Enterobacter sakazakii* meningitis in neonates. Pediatr Infect Dis J. 1988;7:196–9. https://doi.org/10.1097/00006454-198803000-00012

42. Naqvi SH, Maxwell MA, Dunkle LM. Cefotaxime therapy of neonatal gram-negative bacillary meningitis. Pediatr Infect Dis. 1985;4:499–502. https://doi.org/10.1097/00006454-198509000-00012

43. Kleiman MB, Allen SD, Neal P, Reynolds J. Meningoencephalitis and compartmentalization of the cerebral ventricles caused by *Enterobacter sakazakii*. J Clin Microbiol. 1981;14:352–4. https://doi.org/10.1128/JCM.14.3.352-354.1981

44. Burdette JH, Santos C. *Enterobacter sakazakii* brain abscess in the neonate: the importance of neuroradiologic imaging. Pediatr Radiol. 2000;30:33–4. https://doi.org/10.1007/s002470050009

45. Stoll BJ, Hansen N, Fanaroff AA, Lemons JA. *Enterobacter sakazakii* is a rare cause of neonatal septicemia or meningitis in VLBW infants. J Pediatr. 2004;144:821–3.

46. Centers for Disease Control and Prevention (CDC). *Cronobacter* species isolation in two infants—New Mexico, 2008. MMWR Morb Mortal Wkly Rep. 2009;58:1179–83.

47. Broge T, Lee A. A case of *Cronobacter* (*Enterobacter sakazakii*) bacteremia in a breastfed infant. J Pediatric Infect Dis Soc. 2013;2:e1–2. https://doi.org/10.1093/jpids/pit021

48. Ravisankar S, Syed SS, Garg P, Higginson J. Is *Cronobacter sakazakii* infection possible in an exclusively breastfed premature neonate in the neonatal intensive care unit? J Perinatol. 2014;34:408–9. https://doi.org/10.1038/jp.2014.14
